# Rapid Onset Fibrosing Cholestatic Hepatitis C Following Transplant of a Hepatitis C Infected Graft Into a Naïve Recipient

**DOI:** 10.1155/crit/9822131

**Published:** 2026-07-06

**Authors:** Kevin B. Harris, Brian K. Theisen, Qing Chang, Shunji Nagai, Syed-Mohammed R. Jafri, Lejla Jakupovic, Dilip K. Moonka

**Affiliations:** ^1^ Division of Gastroenterology and Hepatology, Henry Ford Health, Detroit, Michigan, USA, henryford.com; ^2^ Division of Pathology and Laboratory Medicine, Henry Ford Health, Detroit, Michigan, USA, henryford.com; ^3^ Division of Transplant and Hepatobiliary Surgery, Henry Ford Health, Detroit, Michigan, USA, henryford.com; ^4^ Division of Inpatient Pharmacy, Henry Ford Health, Detroit, Michigan, USA, henryford.com

**Keywords:** direct-acting antiviral therapy, fibrosing cholestatic hepatitis c, lymphopenia, postliver transplant, sustained virologic response

## Abstract

Fibrosing cholestatic hepatitis (FCH) is a devastating complication that can occur when hepatitis C virus (HCV) recurs following liver transplantation. When it occurs, FCH typically develops between 1 and 6 months following transplant and is associated with markedly elevated HCV levels. With the advent of direct‐acting antiviral therapy, FCH C is now a rare occurrence. Here, we describe a rare case of early‐onset FCH that occurred when an HCV nucleic acid test (NAT) positive liver graft was transplanted into a hepatitis C naïve recipient. This case highlights the importance of initiation of antiviral therapy as soon as possible following transplantation of an HCV NAT‐positive liver graft as recommended by current guidelines.

## 1. Introduction

Historically, hepatitis C virus (HCV) cirrhosis was the leading indication for liver transplantation (LT). Before the advent of direct‐acting antiviral (DAA) therapy for HCV, recurrence of HCV after LT was almost universal. Up to 10% of these patients developed severe HCV recurrence with fibrosing cholestatic hepatitis (FCH). FCH typically occurs between 1 and 6 months after transplant and is associated with jaundice, markedly elevated HCV viral levels, and the absence of biliary or vascular complications [1]. FCH was a potentially devastating complication, in that, it was challenging to treat with interferon‐based therapy and could lead to graft loss and patient death. With the advent of DAA therapy, post‐LT HCV infection can be cured with greater than 95% success. It is now common practice to offer HCV‐naïve patients an HCV‐infected graft and to treat the HCV following transplant [2]. With this approach, the number of available donor organs has increased and FCH is infrequent. In the era of DAA therapy, FCH has been reported only in limited case reports. In 2020, Kapila et al. [3] reported two cases of FCH after kidney transplantation from HCV‐viremic donors to HCV‐negative recipients. More recently, Awan et. al. [4] described the case of a patient who developed FCH following transplantation with an HCV‐positive liver graft. In this case, the patient had concomitant biopsy proven acute cellular rejection and received pulse dose IV methylprednisolone and increased baseline immunosuppression. In our manuscript, we describe a rare case of early‐onset FCH that occurred within days of transplant of an HCV nucleic acid test (NAT) positive liver graft into a hepatitis C naïve recipient. This case is unique as our patient developed FCH while receiving standard immunosuppression which highlights the importance of initiation of antiviral therapy as soon as possible following transplantation of an HCV NAT‐positive liver graft. Written consent was obtained from the patient and donor family before writing this case report.

## 2. Case Presentation

A 29‐year‐old woman with a history of biliary atresia underwent living donor LT at 1 year of age. She did well and was off all immunosuppression but developed extensive multifocal hepatic abscesses following pregnancy. Due to progressive hepatic dysfunction, vascular compromise, and recurrent bacteremia, she was hospitalized at Henry Ford Hospital in Detroit, Michigan in February of 2025. She underwent a donation after brain death (DBD) LT with redo hepaticojejunostomy. She received an HCV NAT‐positive donor organ and received corticosteroids as induction immunosuppression. She was maintained on tacrolimus, mycophenolate mofetil, and prednisone.

**Figure 1:**
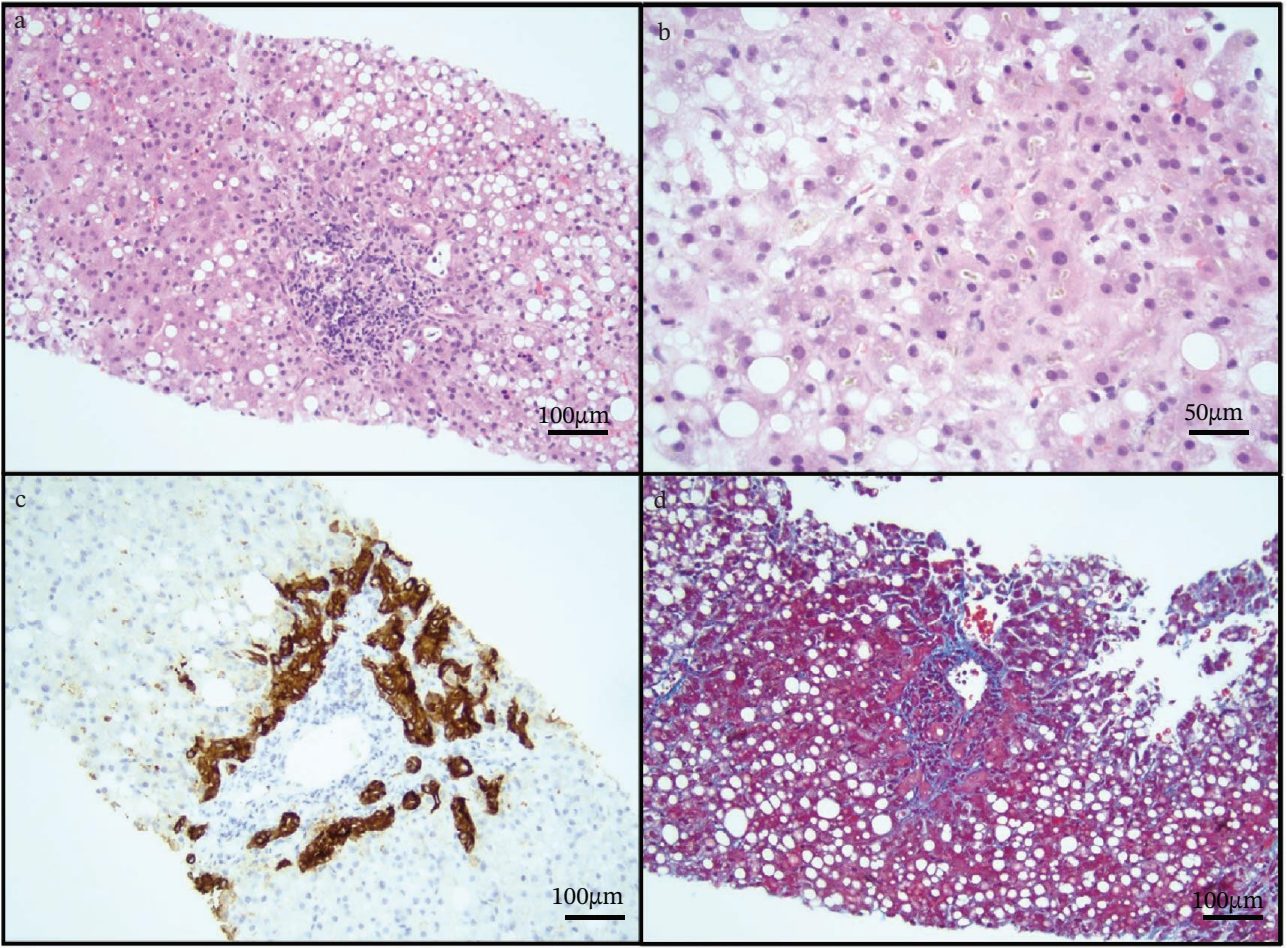
(a) H&E stained sections (10x) demonstrate mild to moderate mixed portal inflammation with cholangiolar reaction in the interface region (highlighted by (c) immunohistochemical stain for CK7 [10x] and (d) pericholangiolar fibrosis on trichrome stain [10x]). Hepatic lobules demonstrate steatosis with mild disarray and occasional swollen hepatocytes. (b) Canalicular cholestasis is noted within hepatic lobules (20x).

On postoperative day 4, she was noted to have increasing liver biochemistries with an HCV viral load of 5,025,050 IU/mL. In the setting of recent bacteremia, she was lymphopenic with an absolute lymphocyte count of 0.22 k/uL (reference range 1.10–4.00 k/uL). On postoperative day 9, her lab showed an ALT 70 IU/mL (reference range ≤ 52 IU/mL), AST 63 IU/mL (reference range ≤ 35 IU/mL), total bilirubin 15.5 mg/dL (reference range ≤ 1.2 mg/dL), and alkaline phosphatase 566 IU/L (reference range 40–140 IU/L) (Table 1). Magnetic resonance cholangiopancreatography revealed no evidence of biliary obstruction or bile leak. The patient underwent a liver biopsy which demonstrated mild to moderate mixed portal inflammation with cholangiolar reaction in the interface region and pericholangiolar fibrosis on trichrome stain. The hepatic lobules demonstrated steatosis with occasional swollen hepatocytes and rare acidophil bodies. There was no convincing portal vein endotheliitis or perivenulitis (Figure 1). The patient′s HCV viral load was 80,547,034 IU/mL with Genotype 2. In the setting of the patient′s high HCV viral load, the biopsy findings were consistent with FCH.

**Table 1:** Laboratory value trend during patient presentation showing worsening liver biochemistries correlating with increasing HCV RNA viral loads. Following initiation of DAA therapy, there is improvement in liver biochemistries without other intervention.

	POD 0	POD 4	POD 9	POD 12	POD 29	POD 49
Clinical event	Liver transplantation		Liver biopsy obtained	DAA therapy started		
ALT (IU/L)	53	91	70	55	51	43
AST (IU/L)	158	48	63	105	31	32
Alkaline phosphatase (IU/L)	33	93	566	558	157	101
Total bilirubin (mg/dL)	3.2	4.4	15.5	22.8	2.5	1.3
Absolute lymphocyte count (K/uL)	0.1	0.22	0.25	0.7	0.6	0.21
Tacrolimus trough level (ng/mL)	0	5.5	11.2	4.5	8.3	9.6
HCV RNA viral level (IU/mL)	Not detected	5,025,050	80,547,034	Not obtained	19	Not detected

Abbreviations: DAA, direct‐acting antiviral therapy; POD, postoperative day.

**Figure 2:**
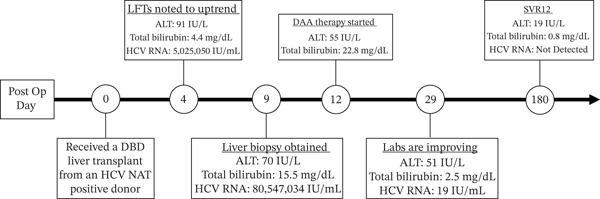
Case timeline from liver transplantation to SVR12.

On postoperative day 12, the patient was started on sofosbuvir/velpatasvir. When treatment was initiated, her total bilirubin was 22.8 mg/dL. The patient′s HCV viral load and liver biochemistries rapidly improved. On postoperative day 29, the patient′s HCV viral load had decreased to 19 IU/mL, and her bilirubin had improved to 2.5 mg/dL. She subsequently achieved sustained virologic response (SVR12) and a normal liver profile (Figure 2). At no point did the patient receive therapy for graft rejection. The patient′s high HCV viral load, liver biopsy findings, and response to antiviral therapy with no other intervention support the diagnosis of post‐LT FCH. The patient is currently 391 days posttransplant and is enjoying excellent graft function with no evidence of recurrent HCV. She continues to follow regularly in post‐LT clinic for routine care.

## 3. Discussion

This case presentation is noteworthy given the rapid development of FCH in a previously HCV‐naïve recipient. Post‐LT FCH has traditionally been described in HCV‐infected recipients, perhaps because they have significant serum viral loads at the time of transplant. In addition, the use of HCV‐infected grafts in HCV‐negative recipients has only found widespread acceptance with the availability of DAA therapy which, in theory, would prevent the development of FCH. In their study advocating for the use of HCV viremic grafts in uninfected patients as “standard of care”, Bohorquez et al. [2] noted they did not see a single case of FCH in 61 uninfected patients receiving infected grafts despite the fact they started antiviral therapy at a mean of 67 days after LT. Therefore, the rapid onset of FCH in this case is remarkable. Typically, with HCV, FCH is seen between 1 and 6 months after LT. Our patient had compelling clinical and histologic evidence of FCH on postoperative day 9. A similar case was reported by Awan et al. where a patient developed FCH on postoperative day 7 following transplantation with an HCV‐positive liver graft into an HCV‐negative recipient [4]. In this case, the patient had concomitant biopsy proven acute cellular rejection and received pulse dose IV methylprednisolone and increased baseline immunosuppression. The authors of that report noted the increased immunosuppression likely exacerbated the HCV infection in their case. Our case is unique as our patient developed FCH while receiving standard immunosuppression. Our patient did have significant lymphopenia from her recent sepsis, which may have increased her risk of developing FCH. A prior report demonstrated that a low peritransplant absolute lymphocyte count is a risk factor for HCV recurrence following LT [5].

Prior reports have described cases of FCH following HCV NAT‐positive kidney and lung transplants into HCV‐naïve recipients [6]. In 2020, two cases of FCH following HCV NAT‐positive kidney transplants were reported. The patients developed FCH at 11 and 14 weeks after transplant and both patients responded to DAA therapy. Similarly, a case of FCH following an HCV NAT‐positive lung transplant into an HCV‐naïve recipient was described. FCH developed several weeks after transplant and responded to treatment with sofosbuvir/velpatasvir/voxilaprevir and ribavirin. Although FCH has been reported in cases where an HCV NAT‐positive donor organ was transplanted into an HCV‐negative recipient, it remains a rare and reportable occurrence.

The current case is informative on several levels. First, it shows that FCH can occur within 10 days of LT in a previously HCV‐naïve patient and that evaluation for jaundice soon after transplant should not focus exclusively on primary graft nonfunction, rejection, or biliary or vascular compromise. This is especially the case in a patient with markedly elevated HCV RNA levels.

Secondly, in accordance with recent practice guidelines, this case argues for the initiation of antiviral therapy as soon as possible following transplant. A prospective multicenter study in 2021 evaluated the kinetics of HCV infection following transplant of D+/R− kidney and LT recipients [7]. In this study, 23 of 24 patients transplanted became viremic after transplant. The median time from transplant to start of antiviral therapy was 7 days for LT patients versus 16.5 days for kidney transplant patients [7]. The median HCV RNA level 3 days after transplant was 6.5 (3.9, 7.1) versus 3.6 (2.9, 4.0) log_10_ IU/mL in LT versus kidney transplant recipients, respectively. At the end of treatment, all of the LT recipients were HCV RNA‐undetectable, whereas three (30%) of the kidney transplant recipients still had detectable, but not quantifiable, viremia. The authors concluded that a preemptive antiviral strategy is effective in treating HCV infection following transplant from an HCV‐positive donor to an HCV‐negative recipient [7]. More recently, the HCV‐TARGET study published in 2024 compared patients that received early (within 7 days of transplant) versus late (after 7 days from transplant) HCV treatment among D+/R− nonliver organ transplants [8]. In this study, patients in the late treatment group followed local treatment algorithms and began HCV therapy at a median of 31 days posttransplant. HCV‐TARGET demonstrated that initiation of DAA therapy within 7 days of transplant leads to fewer virological failures compared to delayed initiation of DAA therapy [8]. According to guidelines from the American Society of Transplantation, HCV viremic donors may be considered for HCV‐negative recipients with informed consent and an established plan to treat HCV posttransplant [9]. The guidelines suggest HCV antiviral therapy may either be preemptive or in the early posttransplant setting after the detection of HCV viremia [9]. Given emerging data that support HCV treatment as early as possible when transplanting an HCV‐viremic liver graft into an HCV seronegative recipient, guidelines from the Infectious Disease Society of America (IDSA) and the American Association for the Study of Liver Diseases (AASLD) recommend initiating HCV therapy within 2 weeks of transplantation, but preferably within 1 week when the patient is clinically stable [10].

Broad implementation of these guidelines from the IDSA and AASLD is lacking in clinical practice. There are several reasons these changes have not been fully implemented. First, access to these medications can be restricted by hospital formularies, insurance coverage, or payer restrictions. Second, there have been prior reports that DAA therapy in the posttransplant setting may lead to immune‐mediated graft dysfunction or an increased risk of acute cellular rejection [11]. The mechanism for this finding is unclear. Although the association between DAA therapy and acute cellular rejection has not been demonstrated across all studies, it has led to some hesitation in starting DAA therapy in the immediate posttransplant setting. Lastly, DAA therapy can interact with tacrolimus, resulting in a significant decrease in tacrolimus concentrations [12]. Patients on DAA therapy and tacrolimus require careful monitoring of their tacrolimus levels to ensure adequate drug trough levels. Despite these concerns, the HCV‐TARGET study found similar rates of rejection in patients that received early HCV treatment (19.6% of patients) versus late HCV treatment (24.5% of patients) [7]. A Cox proportional hazards regression showed no association between graft rejection and the timing of HCV treatment [7].

In a patient able to tolerate oral medication, there is little reason to delay DAA initiation following transplant of an HCV infected organ into a naïve recipient.In our case, DAA initiation was delayed while awaiting insurance approval for inpatient DAA therapy. The transplant community should work to overcome logistical challenges such as insurance approval and DAA access to improve early initiation of these medications following transplant. We have now added antiviral medications for HCV to our inpatient formulary and there may be a role for other centers to do the same.

**Nomenclature**
DAA: direct‐acting antiviral
FCH: fibrosing cholestatic hepatitis
HCV: hepatitis C virus
LT: liver transplantation
NAT: nucleic acid test
POD: postoperative day
SVR: sustained virologic response

## Funding

No funding was received for this manuscript.

## Consent

A statement of written informed consent was obtained from the patient for the publication of case details and use of images. A statement of written informed consent was obtained from the donor′s family for the publication of case details and use of images.

## Conflicts of Interest

The authors declare no conflicts of interest.

## Data Availability

Data sharing is not applicable to this article as no datasets were generated or analyzed during the current study.
